# Cabozantinib Reverses Topotecan Resistance in Human Non-Small Cell Lung Cancer NCI-H460/TPT10 Cell Line and Tumor Xenograft Model

**DOI:** 10.3389/fcell.2021.640957

**Published:** 2021-03-22

**Authors:** Zi-Ning Lei, Qiu-Xu Teng, Pranav Gupta, Wei Zhang, Silpa Narayanan, Dong-Hua Yang, John N. D. Wurpel, Ying-Fang Fan, Zhe-Sheng Chen

**Affiliations:** ^1^Department of Pharmaceutical Sciences, College of Pharmacy and Health Sciences, St. John’s University, Queens, NY, United States; ^2^Institute of Plastic Surgery, Weifang Medical University, Weifang, China; ^3^Department of Hepatobiliary Surgery, Zhujiang Hospital of Southern Medical University, Guangzhou, China

**Keywords:** cabozantinib, non-small cell lung cancer, topotecan resistance, NCI-H460/TPT10, ABCG2, tumor xenograft model, micro-environment

## Abstract

Cabozantinib (CBZ) is a small molecule tyrosine kinase receptor inhibitor, which could also inhibit the ABCG2 transporter function. Therefore, CBZ could re-sensitize cancer cells that are resistant to ABCG2 substrate drugs including topotecan (TPT). However, its reversal effect against TPT resistance has not been tested in a TPT-induced resistant cancer model. In this study, a new TPT selected human non-small cell lung cancer (NSCLC)-resistant cell model NCI-H460/TPT10 with ABCG2 overexpression and its parental NCI-H460 cells were utilized to investigate the role of CBZ in drug resistance. The *in vitro* study showed that CBZ, at a non-toxic concentration, could re-sensitize NCI-H460/TPT10 cells to TPT by restoring intracellular TPT accumulation via inhibiting ABCG2 function. In addition, the increased cytotoxicity by co-administration of CBZ and TPT may be contributed by the synergistic effect on downregulating ABCG2 expression in NCI-H460/TPT10 cells. To further verify the applicability of the NCI-H460/TPT10 cell line to test multidrug resistance (MDR) reversal agents *in vivo* and to evaluate the *in vivo* efficacy of CBZ on reversing TPT resistance, a tumor xenograft mouse model was established by implanting NCI-H460 and NCI-H460/TPT10 into nude mice. The NCI-H460/TPT10 xenograft tumors treated with the combination of TPT and CBZ dramatically reduced in size compared to tumors treated with TPT or CBZ alone. The TPT-resistant phenotype of NCI-H460/TPT10 cell line and the reversal capability of CBZ in NCI-H460/TPT10 cells could be extended from *in vitro* cell model to *in vivo* xenograft model. Collectively, CBZ is considered to be a potential approach in overcoming ABCG2-mediated MDR in NSCLC. The established NCI-H460/TPT10 xenograft model could be a sound clinically relevant resource for future drug screening to eradicate ABCG2-mediated MDR in NSCLC.

## Introduction

Lung cancer remains a leading cause of cancer-related death worldwide (Bray et al., 2018; Siegel et al., 2020). Non-small cell lung cancer (NSCLC) accounts for most of the lung cancer cases with a 5-year survival rate as low as 20% (Osmani et al., 2018). Topotecan (TPT), an FDA-approved anti-cancer drug for small cell lung cancer (SCLC), has exhibited efficacy for advanced NSCLC patients as a first- or second-line treatment (Powell et al., 2013; Vennepureddy et al., 2015). However, resistance to TPT has been shown in NSCLC with multidrug resistance (MDR) phenotype (d’Amato et al., 2007; Li et al., 2017). Cancer MDR refers to the resistance against multiple mechanically and structurally unrelated antitumor drugs in cancer cells, which has become a major contributor to clinical failure in cancer chemotherapy (Ejendal and Hrycyna, 2002; Zhang et al., 2015). The overexpression of ATP-binding cassette (ABC) transporters on the plasma membrane of cancer cells, which efflux an extensive spectrum of chemotherapeutic agents out of the cancer cells, is one of the primary mechanisms contributing to cancer MDR (Sharom, 2008; Zhang et al., 2015). In particular, ABCG2, also known as breast cancer resistance protein (BCRP; Mao and Unadkat, 2015), has been suggested to be closely associated with the resistance against topoisomerase I inhibitors in NSCLC, including TPT, irinotecan, and SN-38 (d’Amato et al., 2007; Fan et al., 2019). To investigate the mechanisms of TPT resistance in NSCLC, we established a TPT-resistant human NSCLC cell line NCI-H460/TPT10. Consistent with the aforementioned hypothesis, overexpression of ABCG2 was observed in the NCI-H460/TPT10 cells compared to their parental NCI-H460 cells, and mechanistic studies indicated that ABCG2 was the major factor conferring TPT resistance in NCI-H460/TPT10 cells (Lei et al., 2020). The established NCI-H460/TPT10 cell line could be a useful model for studying TPT resistance and discovering novel reversal agents to overcome MDR in NSCLC, yet applicability of NCI-H460/TPT10 cell line as a TPT-resistant model *in vivo* has not been verified.

Cabozantinib (CBZ) is a small molecule tyrosine kinase receptor inhibitor (TKI), which targets c-Met and vascular endothelial growth factor receptor 2 (VEGFR2), that has been approved by the FDA to treat advanced renal cell carcinoma and medullary thyroid cancer (Kurzrock et al., 2011; Yakes et al., 2011). In a previous study, the capability of CBZ to inhibit the function of ABCG2 and re-sensitize ABCG2 overexpressing cells to ABCG2 substrate drugs, including TPT, has been demonstrated in *in vitro* settings (Zhang G.N. et al., 2017). Nevertheless, the tests on monolayer-cultured cell models could not mimic the complicated natural tumor micro-environment, which is an important factor affecting tumor growth, metastasis, as well as resistance to therapies *in vivo* (Gillet et al., 2011; Quail and Joyce, 2013). The present study aims to validate the NCI-H460/TPT10 cell line as a clinically relevant model *in vivo* by establishing an NCI-H460/TPT10 tumor xenograft mouse model and to verify the *in vivo* efficacy of CBZ on reversing TPT resistance using the established animal model.

## Materials and Methods

### Chemicals and Reagents

Dulbecco’s modified Eagle’s medium (DMEM), fetal bovine serum (FBS), bovine calf serum (BS), penicillin/streptomycin, and trypsin 0.25% were purchased from Hyclone (GE Healthcare Life Science, Pittsburgh, PA, United States). The radio-labeled [^3^H]-mitoxantrone (4 Ci/mmol) was purchased from Moravek Biochem-icals, Inc. (Brea, CA, United States). Phosphate buffered saline (PBS) and dimethyl sulfoxide (DMSO) were obtained from Thermo Fisher Scientific Inc. (Rockford, IL, United States). Mitoxantrone (MX), SN-38, cisplatin, geneticin (G418), and Ko143 were obtained from Enzo Life Sciences (Farmingdale, NY, United States). CBZ was purchased from Chemietek Company (Indianapolis, IL, United States). The mouse monoclonal antibodies for ABCG2 and glyceraldehyde phosphate dehydrogenase (GAPDH) were obtained from Thermo Fisher Scientific Inc. (Rockford, IL, United States). The rabbit monoclonal antibodies against human DNA topoisomerase I, the HRP-labeled anti-mouse secondary antibody, and the HRP-linked anti-rabbit secondary antibody were purchased from Cell Signaling Technology (Danvers, MA, United States). TPT, 3-(4, 5-dimethylthiazol-yl)-2, 5-diphenyltetrazolium bromide (MTT), and all other chemicals were purchased from Sigma Chemical Co. (St. Louis, MO, United States).

### Cell Lines and Cell Culture

The human NSCLC NCI-H460 cell line was cultured in DMEM containing 10% FBS and 1% penicillin/streptomycin. Its TPT-resistant cell line NCI-H460/TPT10 was maintained in the same culture media supplemented with 10 μM TPT (Lei et al., 2020) and switched into a drug-free medium for more than 2 weeks prior to their use. The ABCG2 gene knockout subline of NCI-H460/TPT10 and the corresponding vector control, which were constructed using clustered regularly interspaced short palindromic repeats (CRISPR)/CRISPR-associated (Cas) 9 system (Lei et al., 2020), were maintained in growth medium supplemented with 1.5 mg/mL G418. The HEK293/ABCG2 and HEK293/pcDNA3.1 cell lines, which were established by transfecting HEK293 cells with the pcDNA3.1 vector containing full-length ABCG2 and the empty vector, respectively, were culture in growth medium containing 2 mg/mL G418. All cell lines were cultured in a humidified atmosphere at 37°C with 5% CO_2_.

### Cytotoxicity Assay

The MTT colorimetric assay was performed to measure the sensitivity of cells to TPT in the presence or absence of CBZ or positive ABCG2 inhibitor Ko143. Briefly, 5 × 10^3^ cells/well were evenly seeded into 96-well plates and cultured overnight. Different concentrations of TPT were added into assigned wells in the presence or absence of CBZ or Ko143 at a fixed concentration. CBZ and Ko143 were added 2 h before TPT. After incubating the plates for 68 h, 4 mg/mL MTT solution was added to each well, and the plates were incubated for an additional 4 h. The medium in each well was subsequently replaced by 100 μL of DMSO. The absorbance at 570 nm was determined by the accuSkan GO UV/Vis Microplate Spectrophotometer. The concentration of CBZ used in the reversal experiment was selected at its IC_20_ (80% of cells remain viable at this concentration) for both cell lines from MTT assay.

### Intracellular Topotecan Accumulation Assay

The intracellular accumulation of TPT in NCI-H460/TPT10 and the parental NCI-H460 cells with or without pre-treatment with CBZ was determined by flow cytometric analysis. Cells (1 × 10^6^/mL) were incubated at 37°C in the culture medium with or without 5 μM CBZ for 2 h followed by an additional 2 h incubation with the culture medium containing 100 μM TPT with or without 5 μM CBZ. At the end of the incubation, cells were washed and resuspended with ice-cold 0.5% bovine serum albumin (BSA) prepared in PBS. The fluorescence from intracellular TPT was analyzed on BD Accuri C6 flow cytometer (BD Biosciences, San Jose, CA, United States) as previously described (Lei et al., 2020).

### [^3^H]-Mitoxantrone Accumulation and Efflux Assay

The effect of CBZ on the intracellular accumulation and efflux of [^3^H]-mitoxantrone was determined in NCI-H460, NCI-H460/TPT10, HEK293, and HEK293/ABCG2 cells. The drug accumulation and efflux assay was performed as previously described (Wang et al., 2020). Briefly, 1 × 10^5^ cells/well were evenly seeded into 24-well plates and cultured overnight. Then, cells were incubated at 37°C in a medium with or without CBZ (5 μM) or Ko143 (5 μM) for 2 h followed by additional 2 h incubation in a medium containing 10 nM [^3^H]-mitoxantrone with or without CBZ or Ko143. After incubation, cells were washed with ice-cold PBS and then incubated in [^3^H]-mitoxantrone-free medium with or without CBZ or Ko143. At various time points (0, 30, 60, and 120 min), cells were harvested and transferred into scintillation fluid. The radioactivity was determined using the liquid scintillation counter (Packard Instrument, IL, United States).

### Western Blotting Analysis

The NCI-H460 and NCI-H460/TPT10 cells were incubated at 37°C for 72 h with vehicle control, 30 nM TPT, 5 μM CBZ, and the combination of 30 nM TPT and 5 μM CBZ, respectively. At the end of incubation, the cell lysates were collected for protein extraction, and the protein expression levels of ABCG2, DNA topoisomerase I (TOP I), and GAPDH were determined using Western blotting analysis. The protein extraction and quantification, gel electrophoresis, and Western blotting analysis were carried out as previously described (Zhang G.N. et al., 2017). A 1:1000 dilution using the blocking agent was applied to all antibodies before use.

### Experimental Animals

Male athymic NCR nude mice (14–16 *g*, age 4–5 weeks) were used for the tumor xenograft model. The project was conducted following the Animal Welfare Act and other federal statutes. The maintenance of the mice and all the *in vivo* studies were conducted in the Animal Care Center of St. John’s University. The animal study was reviewed and approved by the Institutional Animal Care and Use Committee of St. John’s University (Protocol #1925).

### *In vivo* Tumor Model

The TPT-resistant NCI-H460/TPT10 model was modified from the NCI-H460 tumor xenograft model previously established by Chen’s Laboratory (Sodani et al., 2014). A 40 mg/kg oral dose of CBZ was selected based on previous preclinical studies that gave 30 or 40 mg/kg of cabozantinib daily for at least 14 days and showed no remarkable toxicity in mice (Zhou et al., 2016; Koinis et al., 2020). Briefly, 4 × 10^6^ of NCI-H460 cells and 6 × 10^6^ of NCI-H460/TPT10 cells were injected subcutaneously in the same male nude mice, with NCI-H460 and NCI-H460/TPT10 in the left and right flank near the armpit, respectively. The mice were randomized into four groups (6 in each group) after the subcutaneous tumors reached a mean diameter of 0.5 cm. Different groups then received various treatments every 3rd day with a total of 6 times: (1) vehicle solution (10% N-methyl-pyrrolidinone + 90% polyethylene glycol 300) as a negative control by mouth (p.o.); (2) TPT (3.0 mg/kg, i.p.); (3) CBZ (40 mg/kg, p.o.); and (4) combination of TPT (3.0 mg/kg, i.p.) and CBZ (40 mg/kg, p.o.). CBZ was given 1 h before TPT administration. Throughout the study, all mice were weighed, and tumors were measured with a caliper every 3rd day before the treatment. Tumor volumes (V) were calculated as previously described (Tiwari et al., 2013). After the treatment cycle, the mice were euthanized by carbon dioxide, and the tumors were excised and weighed. The ratio of growth inhibition (IR) was calculated according to the formula: IR = 1 – (Mean tumor weight of experimental group/Mean tumor weight of vehicle control group) × 100% (Tiwari et al., 2013).

### Statistical Analysis

One-way ANOVA followed by Tukey’s *post hoc* test was performed for the *in vitro* studies. Two-way ANOVA followed by Tukey’s *post hoc* test was performed for comparing multiple groups with repeated tumor volume measurements in the animal study. Statistical significance was set at *p* < 0.05, and statistical analysis was carried out using GraphPad Prism 8 for macOS (GraphPad Software, La Jolla, CA, United States).

## Results

### The Effect of Cabozantinib on Reversing Topotecan Resistance and Restoring Intracellular Topotecan in NCI-H460/TPT10 Cells

**FIGURE 1:**
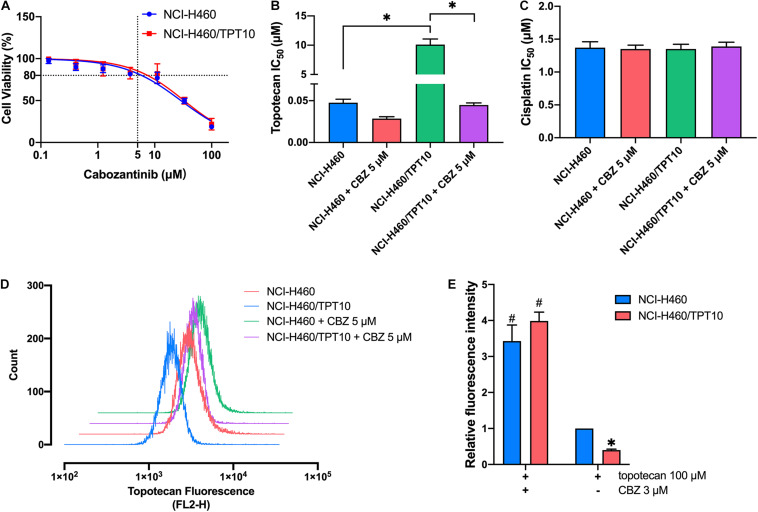
Effect of cabozantinib on reversing topotecan resistance and restoring intracellular topotecan. **(A)** Cell viability was determined by MTT assay and displayed the changes in response to different concentrations of cabozantinib in topotecan-resistant NCI-H460/TPT10 and the parental NCI-H460 cells. Data points with error bars represented the mean viability (%) ±SD of at least three independent experiments, each done in triplicate. **(B,C)** Effects of cabozantinib on the IC_50_ values of topotecan **(B)** and cisplatin **(C)** in NCI-H460/TPT10 and NCI-H460 cells. CBZ is the abbreviation of cabozantinib. Columns and error bars represented mean ± SD of IC_50_ values acquired from three independent experiments in triplicate. ^∗^*p* < 0.05. **(D)** Flow cytometry detection of intracellular accumulation of topotecan in cells after 2 h exposure to 100 μM topotecan with or without 2 h pretreatment with 5 μM cabozantinib. **(E)** Intracellular topotecan accumulations in cells are represented by the fold of fluorescence intensity, which is calculated by (mean FL2-H unit of cells in 100 μM topotecan with or without cabozantinib – mean FL2-H unit of untreated cells with or without cabozantinib)/(mean FL2-H unit of parental cells in 100 μM topotecan – mean FL2-H unit of untreated parental cells). Fluorescence intensity of the accumulated topotecan in NCI-H460 cells without cabozantinib was normalized to 1. Columns and error bars represented average values with SD from three independent measurements. ^∗^indicates *p* < 0.05 comparing resistant cell line to parental cell line with the same treatment; ^#^indicates *p* < 0.05 comparing a group with cabozantinib to the corresponding cell line group without cabozantinib.

Cell-based MTT assay showed that CBZ did not have significant cytotoxicity in NCI-H460 and NCI-H460/TPT10 cells, with an IC_50_ value higher than 10 μM (Figure 1A). Based on this result, CBZ at a concentration of 5 μM, from which 80% of the cells could survive, was selected for reversal studies. As shown in Figure 1B, CBZ, at a non-toxic concentration (5 μM), could significantly decrease the IC_50_ value of TPT in NCI-H460/TPT10 cells. The cross-resistance to other ABCG2 substrates in NCI-H460/TPT10 cells, including mitoxantrone and SN-38, could also be reversed by CBZ with comparable potency to the ABCG2 inhibitor Ko143 (Table 1). On the other hand, the IC_50_ value of cisplatin, which is not a substrate of ABCG2, was not affected by co-administration of 5 μM CBZ (Figure 1C). Furthermore, CBZ could restore TPT accumulation in ABCG2 overexpressing NCI-H460/TPT10 cells (Figures 1D,E). These observations indicated that the CBZ can alleviate TPT resistance most likely by increasing intracellular TPT level, which could be a result from the ABCG2 inhibitory effect of CBZ. A slight reduction of TPT IC_50_ and elevation of TPT accumulation in parental NCI-H460 cells treated with CBZ were observed (Figures 1B,D,E), possibly due to the endogenous ABCG2 expression in NCI-H460 cells.

**TABLE 1:** The drug-resistance of NCI-H460/TPT10 and the reversal effect with cabozantinib.

Treatment	IC_50_ ± SD^a^ (μM; Resistance fold^b)^	
	
	NCI-H460	NCI-H460/TPT10
Topotecan	0.0476 ± 0.0042 (1.00)	10.1044 ± 0.9571 (212.39)*
+CBZ 3 μM	0.0468 ± 0.0041 (0.98)	0.0883 ± 0.0072 (1.86)
+CBZ 5 μM	0.0287 ± 0.0022 (0.60)	0.0449 ± 0.0024 (0.94)
+Ko143 5 μM	0.0292 ± 0.0027 (0.61)	0.0401 ± 0.0042 (0.84)
Mitoxantrone	0.0363 ± 0.0043 (1.00)	4.2621 ± 0.5715 (117.36)*
+CBZ 3 μM	0.0391 ± 0.0022 (1.08)	0.0554 ± 0.0188 (1.53)
+CBZ 5 μM	0.0376 ± 0.0012 (1.04)	0.0373 ± 0.0031 (1.03)
+Ko143 5 μM	0.0325 ± 0.0028 (0.90)	0.0302 ± 0.0042 (0.83)
SN-38	0.0112 ± 0.0020 (1.00)	1.3125 ± 0.1991 (116.96)*
+CBZ 3 μM	0.0131 ± 0.0015 (1.17)	0.0385 ± 0.0052 (3.44)
+CBZ 5 μM	0.0139 ± 0.0022 (1.24)	0.0129 ± 0.0044 (0.98)
+Ko143 5 μM	0.0130 ± 0.0007 (1.16)	0.0188 ± 0.0022 (1.68)
Cisplatin	1.3750 ± 0.0908 (1.00)	1.3575 ± 0.0409 (0.99)
+CBZ 3 μM	1.3883 ± 0.0744 (1.01)	1.3582 ± 0.0722 (0.99)
+CBZ 5 μM	1.3514 ± 0.0591 (0.98)	1.3952 ± 0.0642 (1.01)
+Ko143 5 μM	1.3868 ± 0.0668 (1.01)	1.3765 ± 0.0837 (1.00)

^*a*^IC_50_: concentration that inhibited cell survival by 50% (mean ± SD). ^*b*^Resistance fold represents IC_50_ value for topotecan, mitoxantrone, SN-38, or cisplatin with or without CBZ or Ko143 divided by IC_50_ value for topotecan, mitoxantrone, SN-38, or cisplatin of NCI-H460 cells without CBZ or Ko143. Values in table are determined from at least three independent experiments performed in triplicate. *indicates significant difference from IC_50_ of NCI-H460/TPT10 (*P < 0.05).

### Involvement of ABCG2 in the Reversal Activity of Cabozantinib

**FIGURE 2:**
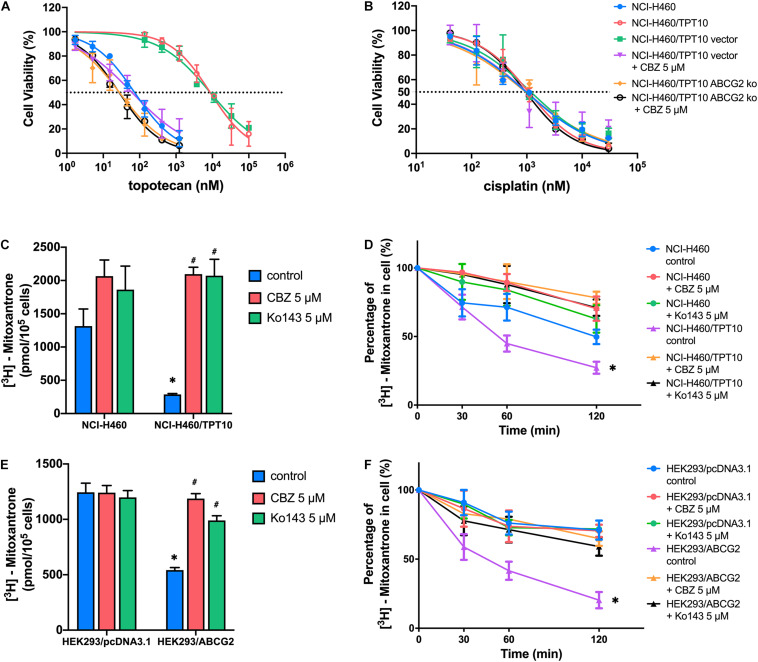
ABCG2 inhibition as a major mechanism for the reversal activity of cabozantinib. **(A,B)** Cell viability was determined by MTT assay and displayed the changes in response to different concentrations of topotecan **(A)** or cisplatin **(B)** with the presence of 5 μM cabozantinib in NCI-H460, NCI-H460/TPT10, the ABCG2 gene knockout subline of NCI-H460/TPT10, and the vector-transfected cells. Data points with error bars represented the mean viability (%) ±SD of at least three independent experiments, each done in triplicate. **(C,E)** The effect of cabozantinib on the cellular accumulation of [^3^H]-mitoxantrone in H460, H460/TPT10, HEK293/pcDNA3.1, and HEK293/ABCG2 cells. ^∗^indicates *p* < 0.05 comparing resistant cell line to parental cell line with the same treatment; ^#^indicates *p* < 0.05 comparing a group with cabozantinib to the corresponding cell line group without ABCG2 inhibitor. Ko143 was used as a positive control of ABCG2 inhibition. **(D,F)** The effect of cabozantinib on the efflux activity of ABCG2 H460, H460/TPT10, HEK293/pcDNA3.1, and HEK293/ABCG2 cells. Data points with error bars represented the mean ± SD of three independent experiments in triplicate. ^∗^indicates *p* < 0.05 compared to NCI-H460 control group or HEK293 control group.

The reversal activity of CBZ was further tested in ABCG2 gene knockout cells derived from NCI-H460/TPT10 cells to further confirm the involvement of ABCG2. The NCI-H460/TPT10-ABCG2 knockout cells restored sensitivity to TPT and was slightly more sensitive compared to NCI-H460 cells that express low level of ABCG2. While CBZ effectively reduced the IC_50_ value of TPT in NCI-H460/TPT10 cells transfected with the vector control plasmid, it failed in further sensitizing NCI-H460/TPT10-ABCG2 knockout cells to TPT (Figure 2A), suggesting that ABCG2 is the key target of CBZ in reversing TPT resistance. As cisplatin was a non-substrate control, the IC_50_ values remained consistent among the cell lines tested regardless of ABCG2 expression level (Figure 2B), which confirmed that the reversal effect of CBZ was specific against ABCG2 substrates.

The results from [^3^H]-mitoxantrone accumulation and efflux assay further verified the aforementioned inference. In consistent to the result from TPT accumulation assay, the intracellular accumulation of [^3^H]-mitoxantrone was significantly higher and the ABCG2 efflux activity was reduced in the NCI-H460 control group, compared to that of the ABCG2 overexpressing NCI-H460/TPT10 cells (Figures 2C,D). CBZ at 5 μM significantly increased the accumulation of [^3^H]-mitoxantrone and mitigated [^3^H]-mitoxantrone efflux, which was comparable to the inhibitory effect on ABCG2 function of 5 μM Ko143. Besides, the intracellular [^3^H]-mitoxantrone level in NCI-H460 cells got a slight rise and the efflux activity was relatively lessened with the presence of CBZ or Ko143, indicating that a low extent of ABCG2 inhibitory effect was exerted to NCI-H460 cells by CBZ or Ko143. Similar reversal effects by CBZ and Ko143 were observed from HEK293/ABCG2 cells (Figures 2E,F). No obvious difference in the intracellular [^3^H]-mitoxantrone accumulation level and efflux activity was shown among HEK293/pcDNA3.1 control, CBZ, and Ko143 group, confirming that the effect of CBZ in increasing drug accumulation and decreasing efflux relied on the expression of functional ABCG2.

### The Effect of Cabozantinib Combined With Topotecan on Protein Expression of ABCG2 and Topoisomerase I

**FIGURE 3:**
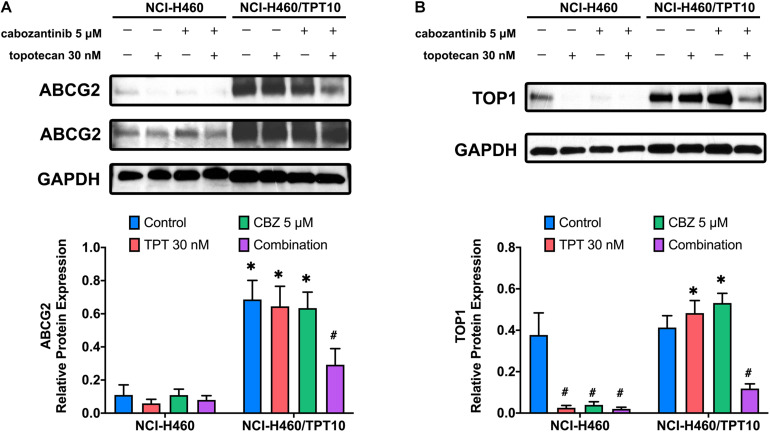
Effect of cabozantinib combined with topotecan on the expression of ABCG2 and topoisomerase I (TOP1). Expression of ABCG2 protein **(A)** and TOP1 protein **(B)** in H460 and H460/TPT10 cell lines treated for 72 h with vehicle control, topotecan (TPT) 30 nM, cabozantinib (CBZ) 5 μM, and combination of topotecan and cabozantinib, respectively, followed by the quantitative analysis. For ABCG2 expression, an imaged obtained with extended exposure time was shown as evidence for the endogenous ABCG2 expression in NCI-H460 cells and was not for quantitative use. The relative protein expression was calculated based on the ratio of target protein versus the loading control protein GAPDH. Columns and error bars represented average values with SD from three independent measurements. ^∗^indicates *p* < 0.05 comparing resistant cell line to parental cell line with the same treatment; ^#^indicates *p* < 0.05 comparing treatment group to control group of the corresponding cell line.

The protein expression levels of ABCG2 and topoisomerase I were investigated to further evaluate the interaction of CBZ with TPT. The low level of endogenous ABCG2 expression in NCI-H460 cells and the overexpression of ABCG2 in NCI-H460/TPT10 cells were verified. As illustrated in Figure 3A, neither CBZ nor TPT alters the ABCG2 protein expression when administrated alone. However, co-administration of 5 μM CBZ and 30 nM TPT downregulated the ABCG2 expression in NCI-H460/TPT10 cells but not in NCI-H460 cells, indicating that the synergistic effect of CBZ and TPT on reducing ABCG2 protein level may be ABCG2-dependent.

As the target of TPT, the topoisomerase I expression in NCI-H460 cells was downregulated by 30 nM TPT monotherapy or combination treatment with 5 μM CBZ. In TPT-resistant NCI-H460/TPT10 cells, the expression of topoisomerase I was not reduced by 30 nM TPT but was significantly reduced by co-administration of 30 nM TPT and 5 μM CBZ (Figure 3B). This is consistent with the cytotoxicity results that resistance to TPT in NCI-H460/TPT10 cells was revered by CBZ and with the TPT accumulation results that the low intracellular TPT level in NCI-H460/TPT10 cells was restored by CBZ. Specially, although the TPT-resistant NCI-H460/TPT10 cell line did not have differential expression of topoisomerase I with the parental NCI-H460 cell line, CBZ specifically downregulates the topoisomerase I expression in the parental NCI-H460 cells.

### The Efficacy of Cabozantinib Combined With Topotecan in NCI-H460/TPT10 Tumor Xenograft Mouse Model

**FIGURE 4:**
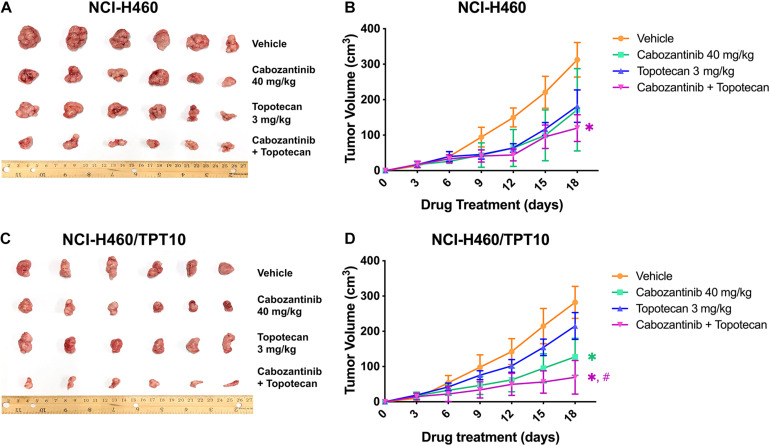
Effect of topotecan and cabozantinib on the growth of NCI-H460 and NCI-H460/TPT10 tumors in male nude mice. **(A,C)** The images of resected tumors at the end of the treatment period from nude mice (*n* = 6 per treatment group) implanted with NCI-H460 and NCI-H460/TPT10 tumors. **(B,D)** The changes in NCI-H460 tumor volume and NCI-H460/TPT10 tumor volume throughout the study after the implantation. Data points and error bars represent the mean and SD of tumor volume (*n* = 6). ^∗^ and ^#^signs were shown in the same color scheme as the figure legends. ^∗^*p* < 0.05 versus the vehicle group; ^#^*p* < 0.05 versus the topotecan only group.

**FIGURE 5:**
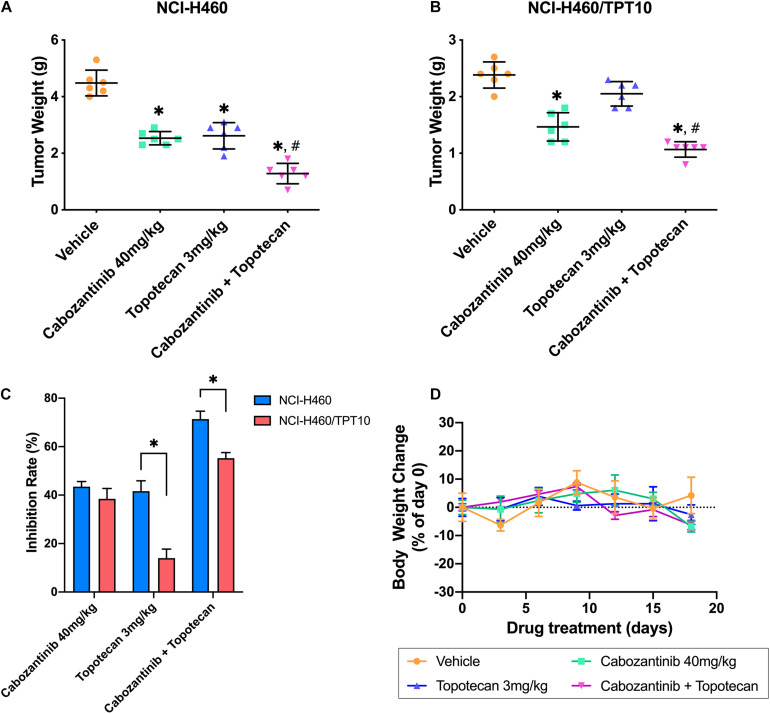
Changes of the tumor weight and the body weight in xenograft mouse model. **(A,B)** Scatter points represented the weights of the excised NCI-H460 tumors and NCI-H460/TPT10 tumors at the end of the 18-day treatment period (*n* = 6 per treatment group). Lines and error bars represented the mean weight values and SD. ^∗^*p* < 0.05 versus the vehicle group; ^#^*p* < 0.05 versus the topotecan group. **(C)** The inhibitory rates of the treatment were demonstrated as the columns with different treatments. IR = 1 – (Mean tumor weight of experimental group/Mean tumor weight of vehicle control group) × 100%. Columns and error bars represented mean and SD. ^∗^*p* < 0.05. **(D)** Body weight changes during the treatment period. Data points and error bars represent the mean and SD of body weight change represented by the percentage of the body weight at day 0 (*n* = 6).

To verify whether the *in vitro* findings could extend to an *in vivo* model, NCI-H460 cells and NCI-H460/TPT10 cells were implanted subcutaneously into athymic nude mice to establish tumor xenograft models. As demonstrated in Figures 4, 5, TPT at 3.0 mg/kg with or without CBZ showed different degrees of anti-cancer activity in tumor xenograft mice without apparent adverse effects or weight loss. TPT alone at 3.0 mg/kg i.p. dose demonstrated significant growth retardation in the drug-sensitive NCI-H460 tumors (Figures 4A,B) but not in the drug-resistance NCI-H460/TPT10 tumors (Figures 4C,D). Similarly, the inhibitory effect of TPT on tumor weight was significantly lower in the NCI-H460/TPT tumors than in the NCI-H460 tumors (Figures 5A–C), suggesting a TPT resistant phenotype in NCI-H460/TPT10 xenograft model. The average tumor volume and tumor weight of implanted NCI-H460 cells and NCI-H460/TPT10 cells were significantly diminished in the CBZ–TPT combination treatment group as compared to the vehicle and TPT alone groups (Figures 4, 5). Besides, the CBZ–TPT combination treatment showed a higher inhibition rate in NCI-H460 xenograft tumors compared to NCI-H460/TPT10 xenograft tumors, indicating possible MDR mechanisms that were not modulated by CBZ in NCI-H460/TPT10 cells *in vivo*. The CBZ alone group also exhibited significant reductions in tumor weight in both cell lines, though the tumor growth inhibitory effect was not as much as that in the CBZ–TPT combination group. These results suggested that the combination of CBZ and TPT could have synergistic effects on both NCI-H460 and NCI-H460/TPT10 xenograft tumors. Overall, the NCI-H460/TPT xenograft model presented the same phenotype of drug-resistance to TPT, and this resistance could be reversed by the ABCG2 inhibitor CBZ in the xenograft mouse model.

## Discussion

It has been suggested that TPT has a good prospect as a first- or second-line treatment for progressed or relapsed NSCLC because of its high efficacy and favorable side effect profile (Vennepureddy et al., 2015). However, as TPT is a substrate of ABCG2, ABCG2-mediated drug exportation could induce TPT resistance in cancer cells, leading to failure in chemotherapy (Mao and Unadkat, 2015). ABCG2 is overexpressed on the plasma membrane of various types of cancer cells functioning as an efflux pump to excrete a broad spectrum of chemotherapeutic drugs (Khunweeraphong et al., 2019). A potential approach to ameliorate Cancer MDR mediated by ABCG2 is co-administration of ABCG2 inhibitors with ABCG2 substrate antineoplastic drugs (Hasanabady and Kalalinia, 2016). Previously, we identified CBZ, a c-Met and VEGFR2 inhibitor, as a novel modulator of ABCG2 with the capability of reversing resistance to mitoxantrone, SN-38, and TPT without affecting ABCG2 protein expression in NSCLC cells (Zhang G.N. et al., 2017). In Zhang et al.’s study, a mitoxantrone-selected MDR cell model that is cross-resistant to TPT due to ABCG2 overexpression was used; however, the effect of CBZ in reversing TPT resistance has not been verified from a TPT-selected resistant NSCLC model. Considering that different biological characters in a TPT-induced resistant model may alter the reversal efficacy of CBZ, in the present study, we utilized our newly-established TPT-resistant human NSCLC cell line NCI-H460/TPT10.

The TPT-resistant NCI-H460/TPT10 cell line was developed from the parental human NSCLC NCI-H460 cell line by stepwise selection with increasing concentration of TPT up to 10 μM (Lei et al., 2020). Analysis of MTT assays in this study showed that the NCI-H460/TPT10 cells retained strong resistance to TPT and cross-resistance to other ABCG2 substrates like mitoxantrone and SN-38 compared to the parental NCI-H460 cells, with resistance folds of 212. 39-, 117. 36-, and 116.96-fold, respectively. And the intracellular TPT accumulation was remarkably lower in NCI-H460/TPT10 cells than in the parental cells. CBZ, acting as a potent ABCG2 inhibitor at its non-toxic concentration (5 μM), could restore the TPT level within NCI-H460/TPT10 cells and significantly re-sensitize NCI-H460/TPT10 to TPT and other ABCG2 substrates, with IC_50_ values comparable to those in the drug-sensitive NCI-H460 cells. Meanwhile, the IC_50_ values of a non-ABCG2 substrate, cisplatin, remained relatively constant between parental and resistant cells with or without CBZ, indicating that CBZ may reduce TPT resistance in NCI-H460/TPT10 cells by the ABCG2 inhibitory mechanism showed in other ABCG2 overexpressing MDR models (Zhang G.N. et al., 2017). Furthermore, CBZ could not sensitize NCI-H460/TPT10-ABCG2 knockout cells to TPT as it did on ABCG2 overexpressing NCI-H460/TPT10 cells, which confirms the involvement of ABCG2 in the reversal activity of CBZ. The inhibitory effect of CBZ on ABCG2 transporting function was further verified by the increased [^3^H]-mitoxantrone accumulation and reduced drug efflux by CBZ in ABCG2 overexpressing NCI-H460/TPT10 cells as well as HEK293/ABCG2 cells. The ABCG2 gene transfected HEK293/ABCG2 cell line and the vector control HEK293/pcDNA3.1 cell line were used to exclude other factors that may be involved in the effect of CBZ on [^3^H]-mitoxantrone accumulation and efflux. As similar results were observed from both pairs of cell lines (NCI-H460 and NCI-H460/TPT10, HEK293/pcDNA3.1 and HEK293/ABCG2, respectively), it was confirmed that CBZ reversed drug resistance by inhibiting ABCG2 efflux function, and ABCG2 may be a major factor responsible for TPT resistance in NCI-H460/TPT10 cells, which are consistent with the previous findings (Lei et al., 2020).

The change in the protein expression level of ABCG2 further revealed the interaction of CBZ with TPT. Consistent to the report from Zhang G.N. et al.’s study (2017), CBZ at 5 μM did not alter the ABCG2 expression in NCI-H460 and NCI-H460/TPT10 cells. However, 5 μM CBZ and 30 nM TPT exhibited synergistic effect in downregulating ABCG2 expression in NCI-H460/TPT10 cells but not in parental NCI-H460 cells, which suggested that the synergistic effect may be dependent on ABCG2 expression. This may be one of the mechanisms accounting for the high toxicity of CBZ and TPT co-administration to NCI-H460/TPT10 cells. The underlying mechanism for CBZ and TPT synergistically downregulating ABCG2 expression and their possible effects on ABCG2-related regulatory factors in ABCG2 overexpressing cells required further study to ascertain. The change of topoisomerase I, which is the target of TPT, was also investigated upon combination treatment of CBZ and TPT. Reduction of topoisomerase I protein expression was observed in NCI-H460 cells treated with 30 nM TPT and the combination of TPT and 5 μM CBZ, as well as in NCI-H460/TPT10 cells treated with the drug combination. It has been reported that TPT could decrease the protein expression of topoisomerase I in ovarian cancer cells and breast cancer cells (D’Onofrio et al., 2011; Parmakhtiar et al., 2019). The downregulation of topoisomerase I expression in NCI-H460 cells upon TPT or combination treatment and in NCI-H460/TPT10 cells upon combination treatment may be explained by the accumulated TPT in NCI-H460 cells and the restored TPT level by CBZ in NCI-H460/TPT10 cells. Interestingly, CBZ downregulated the topoisomerase I expression in the parental NCI-H460 cells but not in NCI-H460/TPT10 cells. It has been found that c-Met expression was positively correlated with topoisomerase I expression in SCLC cells, and inhibition of c-Met could reduce topoisomerase I activity (Rolle et al., 2014). However, considering the topoisomerase I expression levels are similar in NCI-H460 and NCI-H460/TPT10 cell lines, it is less likely that CBZ exerted differential effects on topoisomerase I expression via c-Met inhibition in NCI-H460 cells. In addition, as the concentration of CBZ was non-toxic, the change of topoisomerase I in NCI-H460 cells may not be cell death-related. Nevertheless, it could not be excluded that long-term TPT selection induced topoisomerase I-related gene profile alteration, resulting in irresponsiveness to the regulatory effect of CBZ on topoisomerase I, which needs to be further elucidated.

Although *in vitro* models have been useful tools in studying cancer MDR and developing novel anti-cancer drugs, their direct relevance to clinical cancer cases has been uncertain. Cultured cancer cells that have adapted to the *in vitro* micro-environment that often differs from the actual tumor found in patients, because they do not capture the regulations from the extracellular matrix, cell-matrix interactions, cell–cell interactions in a three-dimensional tumor structure, and the multi-cellular heterogeneous components of the tumor micro-environment such as stromal cells and blood vessels (Asghar et al., 2015). The xenograft animal models based on conventional cancer cell lines have been developed and used for decades to improve the shortage. In order to assess the applicability of the NCI-H460/TPT10 cell line to test MDR reversal agents *in vivo* and to verify the *in vivo* efficacy of CBZ on reversing TPT resistance, a tumor xenograft nude mouse model implanted with NCI-H460 and NCI-H460/TPT10 tumors in the left and right flank near the armpit, respectively, was further established and investigated. In consideration of the clinical relevance of the *in vivo* study, the designed dose of CBZ was evaluated by converting to the corresponding human dose using the method provided by Nair et al. (Nair and Jacob, 2016; Nair et al., 2018). The calculated corresponding human dose for 40 mg/kg mouse dose is 0.813 mg/kg, which is closed to the FDA approved 60 mg/day orally for patients with hepatocellular carcinoma (FDA, 2019), suggesting the potential of the results of this preclinical study to be achieved in the further clinical studies.

The *in vivo* study showed lower TPT efficacy in NCI-H460/TPT10 tumors than NCI-H460 tumors and synergistic anti-cancer effects from CBZ -TPT combination treatment were observed, which verified that the findings of TPT resistant phenotype of NCI-H460/TPT10 cell line and the reversal capability of CBZ in both NCI-H460 and NCI-H460/TPT10 cells could be extended to *in vivo* xenograft models. Similar results were reported by Zhang W. et al. (2017) that a mitoxantrone-selected resistant cell line derived from the NCI-H460 cell line could retain its MDR phenotype and original cytological features after xenografting in athymic nude mice. The NCI-H460 cell line and its drug-resistant sublines may serve as sound models for cancer pharmacology research as they are likely to possess clinically relevant characters such as drug resistance both *in vitro* and *in vivo.* Our results also supported that the NCI-H460/TPT10 cell line could be a favorable model for studying TPT resistance, ABCG2-mediated MDR, and pharmacological evaluations on potential MDR reversal agents.

Interestingly, although our *in vivo* study suggested that the TPT resistance of NCI-H460/TPT10 cells was largely mediated by overexpression of ABCG2, it was observed that the combination of CBZ and TPT had relatively lower efficacy in treating NCI-H460/TPT10 xenograft tumors compared to NCI-H460/TPT10 xenograft tumors, indicating novel MDR mechanisms not being modulated by CBZ in NCI-H460/TPT10 cells *in vivo*, which might be independent to ABCG2 function or expression. In an *in vivo* setting, it is more likely that multiple factors are involved in cancer MDR compared to in a setting of monolayer cell culture with general growth media. Tumor cells can influence the surrounding micro-environment by releasing extracellular signals, promote tumor vascular proliferation and inhibit peripheral immune cells, all these factors can affect the growth and resistance phenotype of tumor cells (Wu and Dai, 2017; Hinshaw and Shevde, 2019). Intra-tumor heterogenicity in the implanted tumors and tumor–host interactions, such as the interplay between the tumors and their micro-environment (Assaraf et al., 2019), may be a factor contributing to the ABCG2-independent MDR observed in NCI-H460/TPT10 xenograft models.

In summary, the reversal effect of CBZ against ABCG2-mediated TPT resistance has been confirmed in the TPT-resistant human NSCLC NCI-H460/TPT10 tumor xenograft model. The established H460/TPT10 cell line and its xenograft model could serve as an invaluable, clinical-relevant resource for future drug screening and the development of novel ABCG2-targeted approaches to eradicate MDR in NSCLC.

## Data Availability Statement

The raw data supporting the conclusions of this article will be made available by the authors, without undue reservation.

## Ethics Statement

The animal study was reviewed and approved by the Institutional Animal Care and Use Committee of St. John’s University.

## Author Contributions

Z-NL, Q-XT, and Z-SC designed the experiments. Q-XT, PG, SN, and WZ performed the experiments. Y-FF, D-HY, and JW provided the technical and material support. Z-SC, Y-FF, and D-HY reviewed and revised the manuscript. All authors discussed the results and implications and commented on the manuscript at all stages.

## Conflict of Interest

The authors declare that the research was conducted in the absence of any commercial or financial relationships that could be construed as a potential conflict of interest.
